# Bioleaching of Transition Metals From Limonitic Laterite Deposits and Reassessment of the Multiple Roles of Sulfur-Oxidizing Acidophiles in the Process

**DOI:** 10.3389/fmicb.2021.703177

**Published:** 2021-07-26

**Authors:** D. Barrie Johnson, Sarah L. Smith, Ana Laura Santos

**Affiliations:** ^1^School of Natural Sciences, Bangor University, Bangor, United Kingdom; ^2^School of Life Sciences, Coventry University, Coventry, United Kingdom

**Keywords:** *Acidithiobacillus*, base metals, biomining, limonite, sulfur, acid dissolution, cobalt, nickel

## Abstract

Using acidophilic bacteria to catalyze the reductive dissolution of oxidized minerals is an innovative process that facilitates the extraction of valuable base metals (principally cobalt and nickel) from limonites, which are otherwise often regarded as waste products of laterite mining. The most appropriate conditions required to optimize reductive mineral dissolution are unresolved, and the current work has reassessed the roles of *Acidithiobacillus* spp. in this process and identified novel facets. Aerobic bio-oxidation of zero-valent sulfur (ZVS) can generate sufficient acidity to counterbalance that consumed by the dissolution of oxidized iron and manganese minerals but precludes the development of low redox potentials that accelerate the reductive process, and although anaerobic oxidation of sulfur by iron-reducing species can achieve this, less acid is generated. Limited reduction of soluble iron (III) occurs in pure cultures of *Acidithiobacillus* spp. (*Acidithiobacillus thiooxidans* and *Acidithiobacillus caldus*) that do not grow by iron respiration. This phenomenon (“latent iron reduction”) was observed in aerated cultures and bioreactors and was independent of electron donor used (ZVS or hydrogen). Sufficient ferrous iron was generated in the presence of sterilized hydrophilic sulfur (bio-ZVS) to promote the effective reductive dissolution of Mn (IV) minerals in limonite and the solubilization of cobalt in the absence of viable acidophiles.

## Introduction

While exploiting microorganisms to extract and recover base and precious metals from mineral ores and wastes (“biomining”) is well established as a global biotechnology, it is currently limited, in commercial-scale operations, to reduced (sulfidic) materials (Johnson, 2014). Some metals that are currently in great demand by industry and domestic consumers are present in significant amounts in oxidized ores, such as nickel where ca. 70% of accessible reserves is deported in laterites (Dalvi et al., 2004). These deposits are widespread in tropical and sub-tropical areas and comprise stratified layers of magnesium-rich saprolite and iron-rich limonite overlying altered ultra-mafic bedrock strata (Butt and Cluzel, 2013). Saprolites typically contain 1.0–2.5% (by weight) nickel and are processed primarily using pyrometallurgy. Limonite layers, which typically contain less nickel but more cobalt than saprolites, are often stockpiled as wastes during mining of laterites, such as in New Caledonia, though some are amenable to high pressure/temperature processing (Stanković et al., 2020). An alternative approach is to use acidophilic microorganisms to extract target metals from limonite at atmospheric pressure and moderate temperatures (30–50°C). Since this process uses acidophilic bacteria to accelerate the dissolution of oxidized minerals by a reductive mechanism, it has been referred to as “biomining in reverse gear” (Johnson and du Plessis, 2015), and developed from earlier discoveries that some facultatively anaerobic acidophiles, such as *Sulfobacillus* spp., were able to catalyze the dissolution of iron (III) minerals such as goethite when provided with a suitable electron donor, in the absence of oxygen (Bridge and Johnson, 1998). Acid leaching of lateritic ores may also be enhanced by addition of various chemical reductants such as sulfur dioxide, dithionite, and some organic acids, as reviewed by Senanayake et al. (2011).

The first reports of successful reductive processing of limonites used pure cultures and consortia of *Acidithiobacillus* spp. that can both oxidize and reduce iron. Metals associated with iron (III) and manganese (IV) oxy-hydroxides (nickel, cobalt, and copper) have been demonstrated to be solubilized by bioleaching limonites from different global locations, using acidophilic bacterial populations (Hallberg et al., 2011; Ñancucheo et al., 2014; Smith et al., 2017). Elemental (zero-valent) sulfur (ZVS) has usually been provided as the electron donor for the microorganisms, which were initially grown aerobically to generate acidity and to build up biomass, before switching to anaerobic conditions by gassing bioreactors with oxygen-free nitrogen (OFN). While this accelerated the rate of mineral dissolution and release of base metals, anaerobic bioleaching required continuous inputs of sulfuric acid to maintain leachate liquors at low pH, as the reductive dissolution of iron (III) and manganese (IV) oxy-hydroxides consumes hydronium ions (Johnson and du Plessis, 2015). Marrero et al. (2015) used a modified approach for bioleaching tailings generated from lateritic ore that had been processed by combined reductive roast and ammoniacal leaching (the CARON process). A pure culture of *Acidithiobacillus thiooxidans*, and a mixed culture of *A. thiooxidans* and *Acidithiobacillus ferrooxidans*, were used to bioleach the tailings in bioreactors that were maintained under aerobic conditions throughout. This significantly reduced the acid-requirement of the process, and 53% of the nickel and 46% of the cobalt were extracted after 11 days using the pure culture. Ferrous iron was reported to accumulate with both cultures with concurrent decline in redox potentials, though the latter was also reported for a bioreactor that was not inoculated. In aerobic environments, *A. ferrooxidans* preferentially oxidizes ferrous iron rather than ZVS (Amouric et al., 2009), and while *A. thiooxidans* cannot use ferric iron directly as an electron acceptor, ferrous iron has long been known to accumulate in oxic cultures of this acidophile grown on ZVS (Brock and Gustafson, 1976).

Here we report a series of experiments in which the relative merits of bioleaching laterite samples (from Kazakhstan and New Caledonia) under aerobic and anaerobic were assessed, and how ferric iron is reduced by *Acidithiobacillus* spp. that cannot respire on iron was investigated. The data obtained have allowed the roles of acidophilic sulfur-oxidizing bacteria in bio-processing limonite to be reassessed, and have suggested how conditions for bio-processing different limonitic samples could be varied to both maximize base metal extraction and minimize costs of consumables.

## Materials and Methods

### Comparative Bioleaching of Limonite Under Aerobic and Anaerobic Conditions

Reductive bioleaching of limonite by a consortium of mesophilic bacteria was compared in two bioreactors maintained under identical conditions but gassed with either sterile atmospheric air or OFN to create and maintain aerobic and anaerobic conditions, respectively. Bioreactor vessels (2 L working volume) coupled to modular units to control temperature, pH, and agitation (Electrolab, United Kingdom) were filled with 1.9 L of a medium (pH 1.8) containing basal salts and trace elements (Ñancucheo et al., 2016), 1 mM ferrous sulfate, and 25 g of ZVS. One hundred milliliters of a consortium of mesophilic acidophiles (ca. 5 × 10^8^ cells mL^–1^) grown in a similar liquid medium, but containing 1 g of ZVS, was added to each bioreactor, both of which were gassed initially with air to promote microbial growth. The consortium contained species of bacteria that were known to couple the oxidation of ZVS to the reduction of either molecular oxygen (under aerobic conditions) or ferric iron (under anaerobic conditions) and also to couple the oxidation of ferrous iron to the reduction of molecular oxygen, and comprised the type strains of *A. ferrooxidans*, *Acidithiobacillus ferriphilus*, *Acidithiobacillus ferridurans*, *Sulfobacillus thermosulfidooxidans*, and *Sulfobacillus acidophilus* (strain BOR1). Once cell numbers had reached ca. 2.5 × 10^8^ cells mL^–1^, the gas supply to one of the reactors was switched to OFN while the second bioreactor continued to be gassed with atmospheric air (both continuous, at ∼ 1 L min^–1^). Limonite ore (100 g, ground to <50 μm) from the Shevchenko deposit in Kazakhstan (Smith et al., 2017; Table 1) was added to both reactor vessels 10 min afterward, and these were stirred at 150 rpm, and maintained at 35°C and at pH 1.8. Samples were withdrawn at regular intervals to measure redox potentials, iron speciation and concentrations of soluble metals over a 20-day experimental period. The volumes of 1 M H_2_SO_4_ and 1 M NaOH required to maintain the bioreactors at pH 1.8 were also recorded.

**TABLE 1:** Elemental* and mineralogical** compositions of the limonite samples used.

	Shevchenko	Penamax		Shevchenko	Penamax
Fe	161	231	Mn	8.8	4.5
Si	213	141	Ni	12	3.0
Al	22	47	Co	1.7	0.42
Ca	11	24	Zn	0.26	0.18
Mg	55	50	Cu	0.03	0.06
Na	3.3	1.0	Ti	0.98	1.42
K	1.7	0.08	V	0.12	0.37
Cr	7.0	8.3	Sc	0.02	0.12

**Shevchenko major minerals*: goethite, maghemite, chromite, smectite, saponite, talc, serpentine, chlorite, quartz, calcite, and asbolane.*

*Penamax major minerals*: goethite, hematite, smectite, glauconite, talc, hornblende, chlorite, quartz, asbolane, and magnetite (trace).

*All concentrations of elements shown are mg kg^–1^. **Mineralogical data are from XRD (X-ray diffraction) and SEM (scanning electron microscopy) analysis.

*All data courtesy of Drs. Agnieszka Dybowska and Paul Schofield (Natural History Museum, London).*

### Bioleaching of Limonite Using Alternation of Gas Supply

To attempt to minimize acid consumption while retaining effective metal solubilization associated the reductive dissolution of oxidized minerals present in Shevchenko limonite, a second experiment was set up using similar conditions to those described above except that the pH was maintained within a wider range (1.75–1.85) which was achieved by alternating the gas supply. When the pH increased to above 1.85, air was pumped into the reactor to promote the microbial generation of sulfuric acid and the pH to fall. When this had decreased to 1.75, OFN was pumped into the vessel to create the anaerobic conditions conducive to reductive mineral dissolution, and concurrent consumption of acidity. Samples were removed and solution chemistry analyzed (as above) over 54 days.

### Bioleaching of Limonite by a Pure Culture of *Acidithiobacillus caldus*^T^ Maintained Under Aerobic Conditions

The type strain of the moderately thermophilic sulfur-oxidizer *A. caldus* was grown in a shake flask containing 1% (w/v) ZVS, basal salts and trace elements, pH 2.0, at 45°C for 1 week. Fifty milliliters of culture (ca. 5 × 10^8^ cells mL^–1^) were then used to inoculate a 2 L bioreactor that contained 12.5 g of ZVS and 1 L of basal salts/trace elements medium (pH 2). The bioreactor was maintained at 45°C, stirred at 150 rpm and gassed continuously with sterile atmospheric air (at about 1 L min^–1^). No pH control was applied in this experiment, and when this had fallen to 1.4 (due to biogenic production of sulfuric acid), 50 g of crushed Shevchenko limonite was added to the reactor. Samples were removed periodically throughout the bioleaching phase of the experiment, which ran for a total of 34 days, and solution chemistry analyzed as above.

### Reduction of Iron and Solubilization of Transition Metals From Limonite in the Absence of Viable Acidophilic Microorganisms

A series of experiments was set up to help elucidate the mechanism(s) of mineral dissolution and ferric iron reduction in acidic liquors containing ZVS, this time using finely ground (<50 μm) limonite from the Penamax mine in New Caledonia (Maurizot et al., 2020; Table 1). In each experiment, the bioreactors were maintained at 50°C, with pH controlled to between 0.9 and 1.0 by automated addition of sulfuric acid. Penamax limonite (50 g) and 1 L of basal salts/trace elements were added to each of the 2 L reactor vessels, which were stirred at 150 rpm but not gassed with either atmospheric air or OFN. In the first experiment, limonite was subjected to acid leaching at 50°C in the absence of added sulfur. Samples were withdrawn at regular intervals to determine redox potentials, soluble base metals, and iron speciation. A second experiment used the same protocol, except that 50 g of sterilized (110°C for 60 min) ZVS powder (VWR chemicals, United Kingdom) was included in the reactor mineral suspension. “Bio-ZVS” (ca. 50 g) was used in a third experiment, which was prepared by transforming hydrophobic ZVS powder to a hydrophilic suspension by including it in an active culture of the mesophilic iron/sulfur-oxidizing mesophilic consortium described above, for 1 week. The “wetted” bio-ZVS was harvested and sterilized (as above) and added to the limonite/basal salts suspension. The liquid basal salts solutions were also sterilized (120°C, 20 min) but the limonite was not, to preclude any changes in mineralogy brought about by treating at high temperatures. In all of these three “abiotic” tests, liquid samples were examined by both microscopic observation and plating on different selective solid media (Johnson and Aguilera, 2016) to test for the presence of viable sulfur- and iron-oxidizing acidophiles at the start and end of the experiments.

### Reduction of Soluble Ferric Iron by *Acidithiobacillus thiooxidans* DSM 103717 During Growth on ZVS, Tetrathionate or Hydrogen as Electron Donor, and by Resting Cells and “bio-ZVS”

In order to elucidate how ferrous iron may be generated and redox potentials lowered during aerobic bioleaching of limonite by *Acidithiobacillus* spp. that cannot grow by ferric iron reduction, as reported previously for both *A. thiooxidans* (Brock and Gustafson, 1976) and *A. caldus* (Johnson et al., 2017) a series of experiments was carried out using a strain of *A. thiooxidans* (DSM 103717, obtained from the DSMZ, Braunschweig, Germany) which was found, unlike the type strain and most others, to be able to use molecular hydrogen, as well as ZVS and various sulfur oxy-anions, as an electron donor for aerobic growth (Falagán and Johnson, 2018). Following adaptation to growing on ZVS, tetrathionate or hydrogen, aerobic ferric iron reduction by cultures of *A. thiooxidans* DSM 103717 was monitored in 50 mL shake flask cultures containing basal salts, trace elements, 50 μM ferrous sulfate and ∼ 5 mM ferric sulfate, supplemented with either 0.5% (w/v) ZVS or 2.5 mM potassium tetrathionate, or incubated in a sealed jar containing H_2_/CO_2_-enriched air (Hedrich and Johnson, 2013). Hydrogen-grown cultures were poised initially at pH 2.0, 1.5, or 1.0. Samples withdrawn at regular intervals to determine concentrations of ferrous iron and to measure optical densities (at 600 nm). Cells grown on either ZVS or hydrogen were also harvested by centrifugation, washed twice and then resuspended (ca. 2 × 10^9^ cells) in 5 mL of sterile basal salts solution (pH 1.5). Sterile ferric iron was added (to ∼ 2.5 mM) and the cell suspensions incubated, with shaking, at 30°C, and changes in concentrations of ferrous iron recorded. Parallel tests using suspension of heat-killed (120°C, 20 min) cells and cell-free spent media were also established.

The reduction of soluble ferric iron by hydrophilic (bio)-ZVS obtained from cultures of *A. thiooxidans* DSM 103717 was also examined. Cultures (100 mL) containing initially 5 g of ZVS were lightly centrifuged (200 × *g*; 1 min) after 10 days to separate solid sulfur from planktonic cells. The ZVS was washed (in pH 1.5 basal salts solution) and centrifuged five further times, and then split into two lots, each containing ∼ 2.5 g of wetted ZVS. Each of these was suspended again in pH 1.5 basal salts (5 mL, total volume), one of which was heat-sterilized while sodium benzoate (100 mg L^–1^, final concentration) was added to the other to act as a biocide. Sterile ferric sulfate was added [∼ 40 mg Fe(III)] to both suspensions, which were subsequently shaken at 30°C, and changes in ferrous iron concentrations recorded over several days.

### Miscellaneous Analytical and Other Techniques

Concentrations of soluble Fe(II) were determined using the Ferrozine assay (Stookey, 1970) and those of other transition metals using a SpectrAA Duo atomic absorption spectrophotometer (Varian, United Kingdom). pH values were measured using a pHase combination glass electrode (VWR, United Kingdom) and redox potentials measurements using a platinum/silver-silver chloride electrode (Thermo Scientific, United Kingdom) and were adjusted to be relative to a standard hydrogen electrode (i.e., *E*_H_ values). Cells were enumerated using a Thoma counting chamber and a phase contrast microscope at ×400 magnification. Mineralogical analysis of fresh and bioleached limonites was carried out at the Natural History Museum, London, using a combination of X-ray diffraction and scanning electron microscopy.

## Results and Discussion

### Bioprocessing of Shevchenko Limonite in Bioreactors Maintained Under Different Physico-Chemical Conditions

**FIGURE 1:**
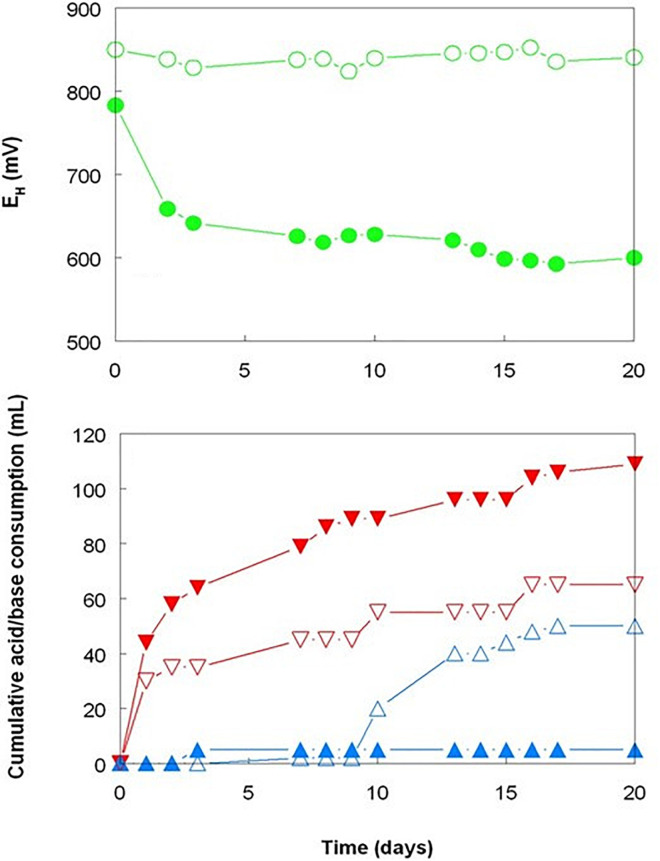
Comparison of redox potentials and acid/base consumption during bioleaching of Shevchenko limonite by a mesophilic consortium of iron-oxidizing/reducing *Acidithiobacillus* spp. maintained at pH 1.8 and 35°C under aerobic (hollow symbols) or anaerobic (filled symbols) conditions. Key: (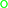, 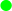) *E*_H_; (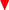, 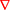) acid; and (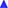, 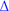) base.

**FIGURE 2:**
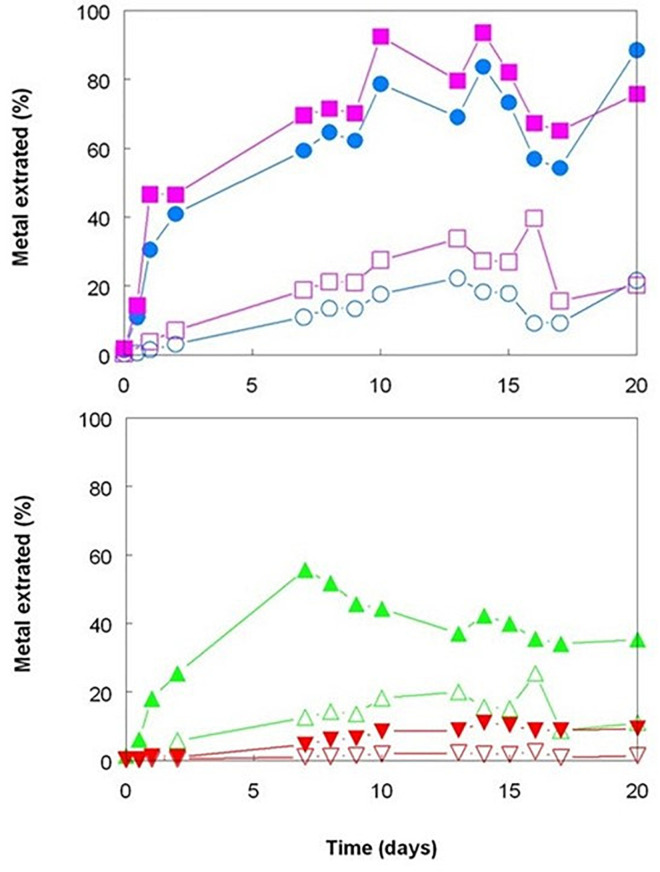
Comparison of base metals solubilized during bioleaching of Shevchenko limonite by a mesophilic consortium of iron-oxidizing/reducing *Acidithiobacillus* spp. maintained at pH 1.8 and 35°C under aerobic (hollow symbols) or anaerobic (filled symbols) conditions. Key: concentrations of total soluble (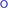, 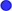) cobalt; (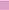, 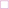) manganese; (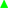, 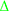) nickel; and (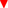, 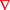) iron.

The three bioreactor experiments carried out with Shevchenko limonite produced data that confirmed, at least to some extent, the results reported by Hallberg et al. (2011) who compared the dissolution of an Australian limonite by reductive acid leaching and acid leaching alone, and by Marrero et al. (2015) who described base metal extraction from laterite tailings from Cuba by cultures of *Acidithiobacillus* spp. under both aerobic and anaerobic conditions. Changing the gas supply from air to OFN in a mesophilic consortium bioreactor resulted in its redox potential falling from just under +800 mV to below +600 mV by day 20, whereas the bioreactor retained under aerobic conditions maintained *E*_H_ values between +830 and +850 mV throughout the experiment (Figure 1). While addition of sulfuric acid was required to maintain both bioreactors at pH 1.8 for the first 9 days following the change in gas supply, this was far greater in the OFN-gassed reactor, and acid demand continued after day 9 for the anaerobic bioreactor in contrast to the aerobic bioreactor which became net acid-producing [i.e., microbiological generation of proton acidity exceeded that consumed in reaction (1); Figure 1]. Much higher concentrations of soluble base metals were found in the anaerobic bioreactor (Figure 2). Those of cobalt and manganese were closely correlated throughout (*r*^2^ = 0.91) and were greater than those of both nickel and iron, while those of soluble nickel peaked at day 7 and declined thereafter (Figure 2) possibly due the formation of secondary mineral phases, such as jarosites. However, although attempts to minimize acid demand by alternating periods of net acid-production (by gassing with air) and net acid-consumption (gassing with OFN, to induce reducing conditions) were accompanied by changes in redox potentials, reflecting net oxidation or reduction of iron, *E*_H_ values did not fall as low as when Shevchenko limonite was bioleached continuously under anaerobic conditions (minimum value +737 mV) and this approach was relatively ineffective at solubilizing the target metals (data not shown).

Acid consumption is an important factor in determining the economic viability of industrial-scale limonite bio-processing, as sulfuric acid is often the greatest consumable cost for the process (Ñancucheo et al., 2014). Reaction (1) indicates that three hydronium ions are consumed for each “FeO.OH” solubilized, though the subsequent bacterial reduction of soluble iron [reaction (2)] generates acidity, lowering net acid-demand:

(1) $$\mathrm{FeO}.\mathrm{OH}+3H_{3}{\text{O}}^{+}↔5H_{2}\text{O}+{\text{Fe}}^{3+}$$

(2) $$6\,{\mathrm{Fe}}^{3+}+{\text{S}}^{0}+11\,H_{2}\,\text{O}\to 6\,{\mathrm{Fe}}^{2+}+{\text{HSO}}_{4}^{-}+7\,H_{3}\,{\text{O}}^{+}$$

Sulfuric acid generated *in situ* by ZVS-oxidizing acidophiles is an attractive alternative to using chemically generated acid, for example to avoid the costs and hazards of transporting concentrated acid to remote mine sites, though aerated bioreactors (and ZVS) would be required. Besides their associated costs, low pH aerobic bioreactors would be highly prone to contamination with iron-oxidizing acidophiles that occupy the same environmental niches as the ZVS-oxidizers (e.g., *Leptospirillum* and *Acidianus* spp.) and which would oxidize any ferrous iron generated. This would eliminate the reducing conditions that helps accelerate the dissolution of oxidized minerals and would result solely in a less effective acid-only leaching process.

**FIGURE 3:**
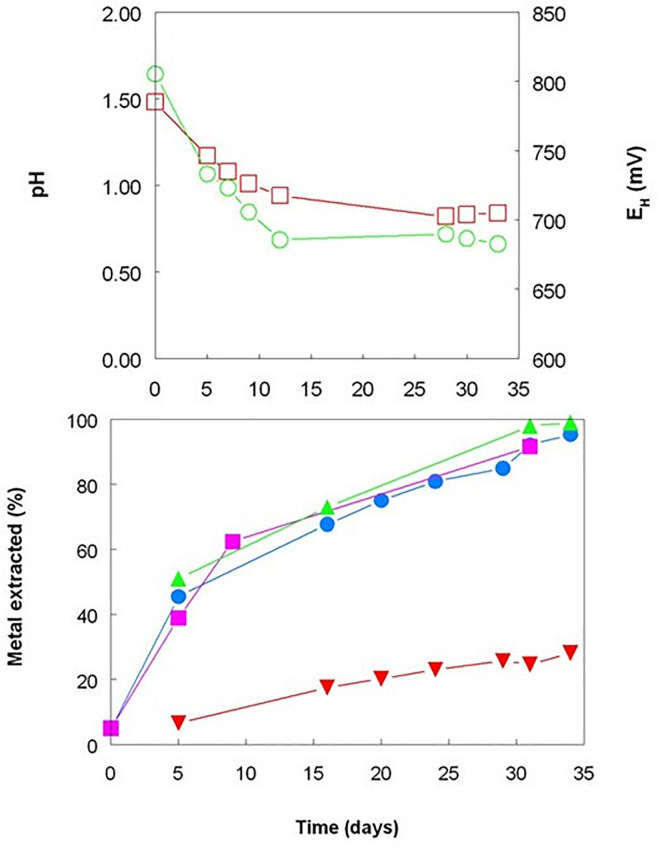
Changes in pH (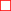) and redox potentials (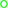), and concentrations of total soluble of cobalt (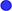), manganese (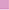), nickel (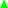), and iron (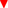) during bioleaching of Shevchenko limonite by a pure culture of *Acidithiobacillus caldus*, maintained at 45°C under aerobic conditions and with no pH control.

In the absence of pH control, the continuous aeration of the *A. caldus* bioreactor caused its pH to decline rapidly, reaching 0.8 (close to the pH minimum for this acidophile; Johnson et al., unpublished data), which would have resulted in more effective acid dissolution of the iron (III) and manganese (IV) minerals present than in the mesophilic bioreactors maintained at pH 1.8 (Johnson and du Plessis, 2015). Some of the solubilized ferric iron was reduced to ferrous, as evidenced by *E*_H_ values becoming less electropositive and ferrous iron concentrations gradually increasing with time. The lowest *E*_H_ recorded in this experiment (+683 mV) was, however, ca.100 mV more positive than that recorded in the first experiment where OFN was used as the gassing phase and about 160 mV more negative than in the aerated bioreactor (Figures 1, 3). This was reflected in concentrations of ferrous and ferric iron, which were present in equimolar concentrations at the end of the experiment using *A. caldus*, whereas >95% of total soluble iron was ferrous in the anaerobic experiment using the mesophilic consortium (the *E*^0^ of the ferrous-ferric couple varies between +655 and +685 mV in extremely acidic sulfate-rich liquors; Johnson et al., 2017). Although *A. caldus* cannot respire on iron, reduction of soluble ferric iron in aerobic cultures has previously been reported for this acidophile (Johnson et al., 2017) as it had been for the related species *A. thiooxidans* (Brock and Gustafson, 1976). The question of how these bacteria mediate iron reduction in the presence of oxygen, and the potential importance of this for bioprocessing limonite deposits, is discussed below. While cobalt, manganese and nickel were effectively bioleached from Shevchenko limonite in the *A. caldus* bioreactor, with over 90% of all three metals solubilized by day 34 (Figure 3), this was initially, in the case of both cobalt and manganese, though not of nickel, a slower process than that achieved by the facultatively anaerobic, mesophilic consortium maintained at higher pH. In contrast to the mesophilic consortium maintained at pH 1.8, no reprecipitation of solubilized nickel and other transition metals was observed in the *A. caldus* bioreactor, probably as a result of the lower pH achieved which would have minimized the generation of secondary minerals such as jarosites. Previously, Marrero et al. (2015) showed that the solubilization of both cobalt and nickel was greater in the presence of *A. thiooxidans* than in non-inoculated controls, although the pH of their inoculated aerated bioreactors increased (in contrast to the current report which used *A. caldus*) and redox potentials declined significantly, which would not be anticipated, in the control vessels.

### Solubilization of Transition Metals From Penamax Limonite in the Absence of Viable Acidophilic Microorganisms

**FIGURE 4:**
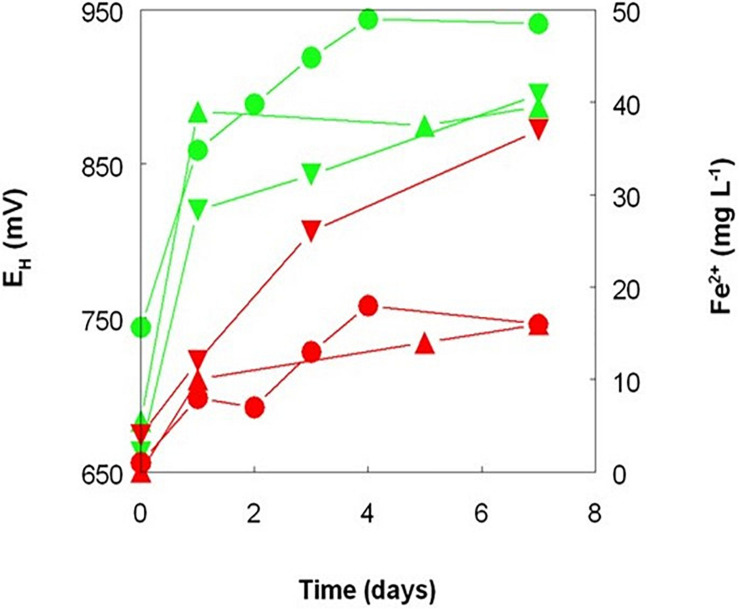
Changes in redox potentials (green symbols) and concentrations of ferrous iron (red symbols) during leaching of New Caledonian limonite at 50°C and pH 1.0, in the presence of sterile sulfur powder (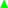, 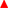), bio-ZVS (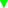, 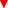), and no added sulfur (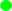, 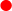).

**FIGURE 5:**
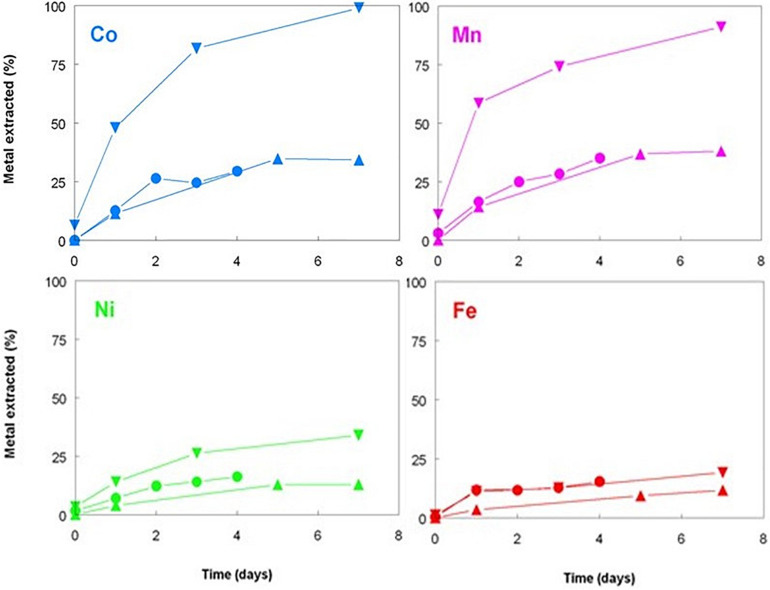
Changes in concentrations of base metals during leaching of New Caledonian limonite at 50°C and pH 1.0, in the presence of sterile sulfur powder (▲), bio-ZVS (▼), and no added sulfur (•).

Elemental sulfur is the preferred electron donor used to promote the bacterial reductive dissolution of limonite due its relatively low cost and lower net acid consumption than when using organic electron donors, such as glycerol (Hallberg et al., 2011; Johnson and du Plessis, 2015). Tests carried out with Penamax limonite in low pH stirred reactor vessels that contained heat-sterilized bio-ZVS that were not gassed with either air or OFN showed that it was possible to enhance the solubilization of significant amounts of base metals in the absence of metabolically active acidophilic bacteria, suggesting a possible role of bio-ZVS itself and/or heat-killed cells in mediating iron reduction. Redox potentials increased rapidly to ∼ +900 mV in all three abiotic reactors (and to the greatest extent in the ZVS-free reactor; Figure 4) reflecting the large amount of ferric iron that was acid-leached from the deposit (1.3–2.0 g L^–1^ by day 7). Smaller concentrations of ferrous iron were also detected in the leachates, accounting for between 0.8 and 2.0% of total soluble iron and were notably greater in the reactor containing bio-ZVS than in the other two (Figure 5). These data suggested that acid dissolution released some ferrous iron directly from the limonite and also that some of the solubilized ferric iron was also being reduced in the presence of bio-ZVS. No viable acidophilic microorganisms were observed in microscopic examination of leachates or isolated on selective solid media in all three abiotic experiments. Changes in concentrations of base metals in this experiment are shown in Figure 5. Acid extraction alone and in the presence of non-wetted ZVS produced similar results, but far more base metals were solubilized in the presence of bio-ZVS. This was particularly notable in the cases of cobalt and manganese (which were again closely correlated; *r*^2^ = 0.97) with 99 and 90% of these metals, respectively, being leached from the limonite by day 7. The implication that ferric iron was being reduced in the presence of bio-ZVS helps explain this observation, as the ferrous iron generated would have had a direct role in mediating the reductive dissolution of Mn (IV) minerals (Johnson and Pakostova, 2021) and the release of associated cobalt and nickel. Many lateritic-limonites contain relatively little cobalt, and this is chiefly associated with asbolane and other Mn (IV) minerals so that even relatively small amounts of ferrous iron can result in significant percentages of Mn and Co being extracted. In contrast, most nickel in limonites is associated with ferric oxy-hydroxide minerals, such as goethite (Johnson and du Plessis, 2015). The Penamax limonite used in the experiment described contains 0.44% (by weight) of manganese and 0.04% cobalt (Table 1). Assuming that all of the manganese was present in oxidized minerals such as asbolane, ∼ 400 mg of ferrous iron would have been required to cause the reductive dissolution of 90% of this mineral phase under the experimental conditions used, given that two ferrous irons are required to reduce one manganese (IV) to manganese (II). The leachate liquor in the bio-ZVS-amended reactor contained ∼40 mg of residual ferrous iron and ∼200 mg manganese (II) after 7 days, implying that the total amount of ferrous iron that had been generated via ferric iron reduction and/or released from the limonite by acid dissolution was ∼440 mg. The contribution of the latter to the net ferrous iron present can be estimated by considering the mass balance of iron and manganese in the reactor that contained no added sulfur, where there was no mechanism for reducing the ferric iron released from limonite by acid dissolution. In this case, 80 and 20 mg of manganese (II) and ferrous iron, respectively, were present in the reactor liquor after 7 days, implying that 180 mg of ferrous iron had been released directly from the limonite. This was ∼40% of the theoretical amount in the reactor that contained bio-ZVS and equivalent to ∼1.5% of the total iron present in the limonite. Minerals that contain iron (II) (chlorite, hornblende and magnetite) were identified in the non-leached Penamax limonite (Table 1) and no chlorite was detected in Penamax limonite that had been (bio)leached in acidic liquors. Minor amounts of manganese (II) were also likely to be present in the chlorite, hornblende and asbolane fractions. In addition, manganese (IV) oxides may undergo limited acid dissolution, forming soluble manganese (IV) hydroxyl complexes [Mn(OH)_4_ and Mn(OH)_3_^+^; Swain et al., 1975].

### Reduction of Soluble Ferric Iron by *A. thiooxidans* DSM 103717 During Aerobic Growth on Different Electron Donors, and by Resting Cells and Bio-ZVS

Ferric iron was reduced to ferrous in cultures of *A. thiooxidans* DSM 103717 growing aerobically with ZVS, tetrathionate or hydrogen as electron donor. In the case of tetrathionate, 1.71 ± 0.45 mM ferric iron was reduced in cultures that originally contained 2.5 mM S_4_O_6_^2–^, which may be accounted for by the transient appearance of thiosulfate. *Acidithiobacillus* spp. produce tetrathionate hydrolase (TetH), which is located outside of the cytoplasm and converts tetrathionate to sulfate and disulfane monosulfonic acid (−S-S-SO_3_; Dahl, 2020). The latter compound is highly reactive and decomposes spontaneously to ZVS, and thiosulfate which is oxidized by ferric iron, regenerating tetrathionate [reaction (3)]:

(3) $$2\,S_{2}\,{\text{O}}_{3}^{2-}+2\,{\mathrm{Fe}}^{3+}\to {\text{S}}_{4}\,{\text{O}}_{6}^{2-}+2\,{\mathrm{Fe}}^{2+}$$

Concentrations of ferrous iron increased, on average, from 0.38 to 2.13 mM, 10 days after 5 mM ferric iron was added to cultures growing aerobically on ZVS. How this was mediated is, however, less clear. While thiosulfate has been suggested to be involved (Marrero et al., 2020) it is counter-intuitive that these bacteria would exude a readily metabolized sulfur oxy-anion generated within the cytoplasm from homocyclic S8 (Wang et al., 2019) and no iron reduction was observed in cell-free media of ZVS-grown cultures (data not shown). Metabolism of ZVS by *Acidithiobacillus* spp. is thought to first involve attachment of the bacteria to sulfur particles by way of glycocalyx-like extracellular polymeric substances, followed by activation of ZVS (involving thiols of specific outer-membrane proteins) and transportation into the cytoplasm as persulfide sulfane-sulfur (Dahl, 2020). This “activated” sulfur is a possible candidate for the observed reduction of soluble ferric iron.

**FIGURE 6:**
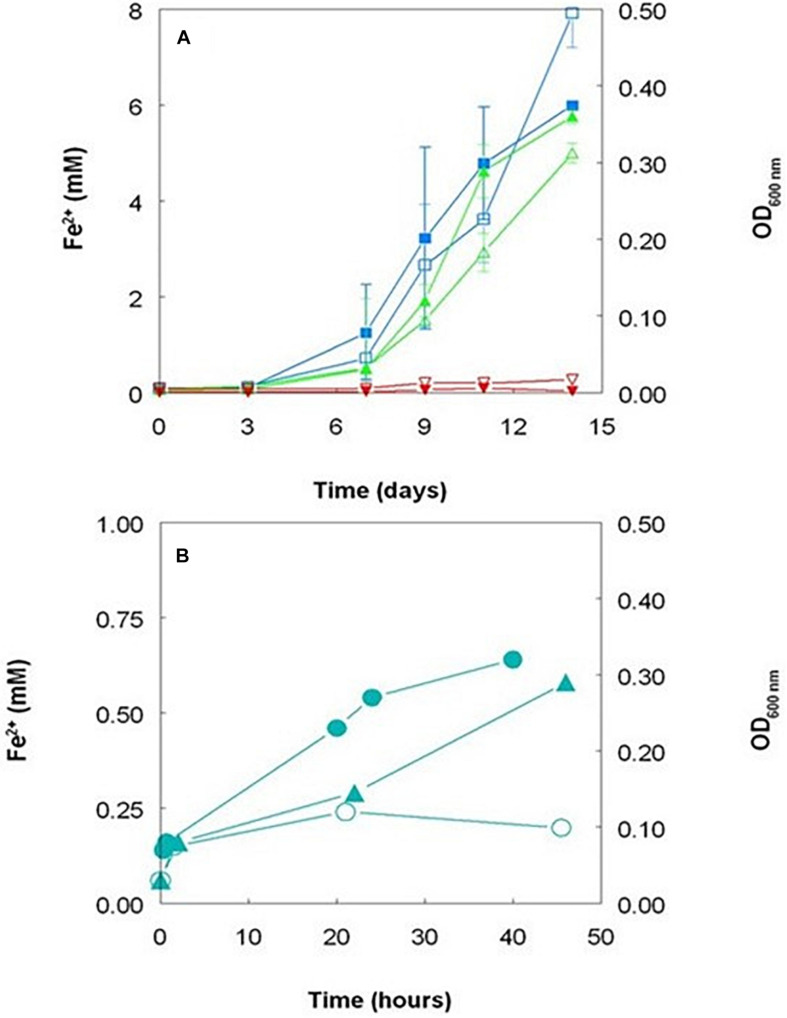
**(A)** Changes in concentrations of ferrous iron (filled symbols) and culture optical densities at 600 nm (hollow symbols) in aerobic cultures of *Acidithiobacillus thiooxidans* DSM 103717 grown at pH 2.0 (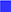, 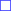), 1.5 (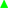, 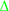), and 1.0 (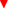, 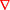); **(B)** changes in concentrations of ferrous iron in harvested and washed cell suspensions of *Acidithiobacillus thiooxidans* DSM 103717 grown on hydrogen (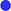) or ZVS (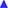), and by heat-killed cell suspensions grown on hydrogen (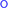). The hydrogen-grown suspensions contained ca. 8.5 × 10^8^ cells mL^–1^ and the ZVS-grown suspensions ca. 5.2 × 10^8^ cells mL^–1^.

However, the fact that iron reduction by *Acidithiobacillus* spp. that do not respire on ferric iron does not necessarily involve thiosulfate or any other sulfur intermediate was confirmed using cultures grown aerobically on hydrogen as electron donor where the only form of sulfur present was sulfate. Data (Figure 6A) show that ferric iron reduction occurred more rapidly in cultures initially poised at pH 2.0 than at pH 1.5, and changes in ferrous iron concentrations were much smaller in pH 1.0 cultures. Iron reduction paralleled growth (as determined by optical density measurements) in hydrogen-grown cultures, and OD values continued to increase after all of the ferric iron added had been reduced. pH values had declined to some extent after 14 days in cultures poised initially at pH 2.0 (to pH 1.7 ± 0.04) and pH 1.5 (to pH 1.42 ± 0.02) but not in pH 1.0 cultures, as reduction of soluble ferric iron is an hydronium ion-generating reaction. *Acidithiobacillus* spp. that do not respire on ferric iron can therefore reduce ferric iron irrespective of electron donor and even whether cells are metabolically active or resting, a phenomenon we refer to as “latent iron reduction.” Why these bacteria reduce iron is unclear, as it appears to have no obvious metabolic function, though one reason might be because ferric iron is often more toxic to acidophilic bacteria than ferrous iron (e.g., Falagán et al., 2019).

Ferric iron reduction was also mediated by harvested cells of *A. thiooxidans* DSM 103717 that had been grown using either ZVS or hydrogen as electron donor, though to a more limited extent than observed in actively growing cultures (Figure 6B). As was the case with ZVS, no reduction of ferric iron was observed in cell-free media of cultures grown on hydrogen as electron donor. A small amount of reduction of ferric iron was observed in the presence of heat-killed cells (Figure 6B) and this may have contributed to the generation of ferrous iron in the “abiotic” bioleaching of Penamax limonite where heat-sterilized bio-ZVS was added (Figure 4). Some reduction of iron was also observed in suspensions of bio-ZVS where attempts had been made to remove bacterial cells from the hydrophilic sulfur ahead of heat-sterilization or suppressing bacterial activity by adding benzoate, though only small amounts of soluble ferric iron were reduced in both cases, with ferrous iron concentrations increasing from 60 to 210 μM over 115 h, both in benzoate-containing suspensions, and from 3 to 80 μM over 70 h with heat-treated bio-ZVS.

### Reappraisal of the Roles of Acidophilic Bacteria in Bioleaching Limonitic Laterites, and Implications for Pilot- and Full-Scale Operations

Data from the current study and previous reports suggest that *Acidithiobacillus* spp. have at least four potential roles in the reductive bioleaching of limonite: (i) generating (sulfuric) acid, by coupling the oxidation of ZVS to the reduction of oxygen (all species) or soluble ferric iron (iron-oxidizing/reducing species only); (ii) generating and maintaining low redox potentials in oxygen-free mineral suspensions (iron-oxidizing/reducing species only); (iii) latent reduction of ferric iron in aerated mineral suspensions (all species); (iv) generating a more reactive form of sulfur, by combined “wetting” and potential formation of sulfane derivatives. Limonite deposits, even from within a single geographical area, can display considerable variations in how amenable they are to bio-processing (Santos et al., 2020) and testing and fine-tuning of the main operational parameters (pH, *E*_H_ and temperature) is required to identify optimum conditions for bioleaching and to determine which of the specific microbial roles in this (e.g., acid production or iron reduction) should be prioritized, and how operational protocols (e.g., pH, temperature, and aeration status) should be engineered to optimize these.

## Conclusion

Acidophilic sulfur-oxidizing bacteria have multiple roles in the bio-processing of oxidized limonitic ores, including novel involvement, such as the transformation of hydrophobic ZVS into more reactive hydrophilic ZVS, that were revealed for the first time in the present study. Dependent on the mineralogy of the limonite deposit, such as the relative amount of acid-labile ferrous iron minerals such as chlorite that might be present, leaching using biogenic acid generated under aerobic conditions can be sufficient to solubilize much of the cobalt present and associated with manganese (IV) minerals. In contrast, microbially mediated ferric iron reduction is more important in promoting the dissolution of iron (III) minerals and the solubilization of nickel. Maintaining low pH (<1) can accelerate the process and diminish the reprecipitation of solubilized transition metals. *Acidithiobacillus* spp. that can use ferric iron as an electron acceptor in the absence of oxygen are particularly prolific at lowering redox potentials and accelerating reductive mineral dissolution. More limited “latent” iron reduction is observed with other species (*A. thiooxidans* and *A. caldus*) though this occurs in pure cultures that are incubated aerobically, concurrent with the genesis of sulfuric acid. Aerobic iron reduction by *A. thiooxidans* DSM 103717 was found not to be dependent on the electron donor used (ZVS, tetrathionate, or hydrogen) and is therefore not necessarily mediated, as previously considered, by a sulfur intermediate.

## Data Availability Statement

The raw data supporting the conclusions of this study are available by request to DBJ at the following email address: d.b.johnson@bangor.ac.uk.

## Author Contributions

DBJ: experimental work, writing manuscript, and preparing figures and tables. ALS: experimental work (Penamax limonite), conceptual discussions, and editing manuscript. SLS: experimental work (Shevchenko limonite). All authors contributed to the article and approved the submitted version.

## Conflict of Interest

The authors declare that the research was conducted in the absence of any commercial or financial relationships that could be construed as a potential conflict of interest. The handling editor declared a past co-authorship with one of the authors DBJ.

## Publisher’s Note

All claims expressed in this article are solely those of the authors and do not necessarily represent those of their affiliated organizations, or those of the publisher, the editors and the reviewers. Any product that may be evaluated in this article, or claim that may be made by its manufacturer, is not guaranteed or endorsed by the publisher.
